# Management of chronic Hepatitis C at a primary health clinic in the high-burden context of Karachi, Pakistan

**DOI:** 10.1371/journal.pone.0175562

**Published:** 2017-04-27

**Authors:** Yuely A. Capileno, Rafael Van den Bergh, Dmytro Donchunk, Sven Gudmund Hinderaker, Saeed Hamid, Rosa Auat, Gul Ghuttai Khalid, Razia Fatima, Aashifa Yaqoob, Catherine Van Overloop

**Affiliations:** 1 Medecins sans Frontieres, Brussels, Belgium; 2 International Union Against Tuberculosis and Lung Disease, Paris, France; 3 University of Bergen, Bergen, Norway; 4 The Aga Khan University and Hospital, Karachi, Pakistan; 5 National TB Control Program, Islamabad, Pakistan; Chiba University, Graduate School of Medicine, JAPAN

## Abstract

**Background:**

The burden of hepatitis C (HCV) infection in Pakistan is among the highest in the world, with a reported national HCV prevalence of 6.7% in 2014. In specific populations, such as in urban communities in Karachi, the prevalence is suspected to be higher. Interferon-free treatment for chronic HCV infection (CHC) could allow scale up, simplification and decentralization of treatment to such communities. We present an interim analysis over the course of February-December 2015 of an interferon-free, decentralised CHC programme in the community clinic in Machar Colony, Karachi, Pakistan.

**Design:**

A retrospective analysis of a treatment cohort.

**Results:**

There were 1,089 patients included in this analysis. Aspartate to platelet ratio index score was used to prioritize patients in terms of treatment initiation, with 242 patients placed in high priority for treatment and 202 starting treatment as scheduled. 169 patients started HCV treatment with Sofosbuvir-Ribavirin regimen according to HCV genotype over the course of 2015: of these, 35% had Hemoglobin reductions below 11.0 g/dl during the treatment course. Among the 153 patients (85%) with genotype 3 HCV infection, 84% of patients achieved sustained virologic response at 12 weeks following treatment completion (SVR 12).

**Conclusion:**

Outcomes of HCV treatment with all oral combination in an integrated, decentralized model of care for CHC in a primary care setting, using simplified diagnostic and treatment algorithms, are comparable to the outcomes achieved in clinical trial settings for Sofosbuvir-based regimens. Our results suggest the feasibility and the pertinence if including interferon-free treatment regimens in the national programme, at both provincial and national levels.

## Introduction

Hepatitis C Virus (HCV) infection is an urgent global health concern. The World Health Organization (WHO) estimates that more than 185 million people are infected with HCV. [1] Transmission is blood-borne, occurring through unsafe injection practices, inadequate sterilization of instruments, blood transfusion, sexual transmission, and mother-to-child transmission. [1] Chronic Hepatitis C can develop into cirrhosis and hepatocellular carcinoma, and ~350,000 people are estimated to die from these complications annually. [2] The prevalence of HCV infection varies worldwide: the Middle East and North African regions, including Egypt and Pakistan, register among the highest prevalence of HCV. [3]

Pakistan is a lower middle-income country with a population of approximately 180 million. [4] In 2014, an estimated adult HCV seroprevalence of 6.7% was reported in Pakistan. [4,5] The province of Sindh, where Karachi is situated, has a recorded seroprevalence of 5.5% among the general population. [6,7] Most important risk factors for HCV transmission in Pakistan are health system-related, including a documented high frequency of therapeutic injections, [8] reuse of syringes, and unlicensed clinics conducting high volumes of blood transfusions, dental surgeries, etc. [9] Most HCV infections in Pakistan are genotype 3 (69.1%), followed by genotypes 1 (7.1%), 2 (4.2%) and 4 (2.2%). [5,7] Interferon (IFN)-based treatment for HCV is recommended by the Chief Minister’s Programme for Hepatitis B and C, and a 67% end of treatment viral clearance for such regimens has been documented. [10] HCV treatment is conventionally offered through specialized, tertiary care-level governmental hospitals. Given the high-burden of the disease, these centers are overwhelmed with patients. [11]

Médecins Sans Frontières (MSF), an international medical humanitarian organization, together with the local nongovernmental organization SINA operates a primary health clinic in Machar Colony, one of the biggest slums in Karachi, with a high prevalence of key risk factors for Hepatitis C. The clinic has ~13,000 monthly patients from the OPD consultations, Expanded Program of Immunization services, delivery unit and health promotion services. Since February 2015, to ensure access to quality HCV treatment for the residents of Machar Colony, the MSF programme has delivered HCV treatment integrated in a primary health care center, using direct acting antivirals (DAAs), in accordance with the current and updated international guidelines on HCV management. Available data on CHC treatment in Pakistan describes outcomes of IFN-based therapy, but no published data on programme outcomes of CHC treatment using IFN-free DAA regimens was available as of November 2016. Likewise, performance indicators such as pre-treatment attrition and end of treatment outcomes for HCV care integrated at primary health care level have not often been documented. In this work, an interim analysis of treatment outcomes of patients enrolled in the HCV programme from February–December 2015 was done. Specifically, patient demographics and clinical characteristics, uptake of care, and adverse outcomes are discussed.

## Methods

### Design

This retrospective cohort study was done among CHC patients enrolled in the Hepatitis C programme at the MSF-SINA Machar Colony clinic in Karachi, Pakistan.

### Setting

Karachi is the capital city of the Province of Sindh, and in 2013 had a population of ~23.5 million. It is the main seaport and financial center of Pakistan. [12] Machar Colony is located in the southwest of the city near the fish harbor just south of Lyari town, one of the more unsecure parts of Karachi. With an estimated population of 700,000, it’s one of the biggest non-regularized slums in the city. [13]

Patients attending the MSF clinic and meeting the screening criteria (see Table 1) are screened using a rapid diagnostic test for HCV antibody (OraQuick). Once tested positive, HCV infection is confirmed through qualitative PCR. Patients coming to the clinic with a documented Chronic Hepatitis C based on HCV RNA PCR by another actor are also considered eligible for care. Positive patients (both those diagnosed in the programme and those with an external diagnosis) are then enrolled in care and assessed for treatment eligibility using the aminotransferase/platelet ratio index (APRI) score, a noninvasive serum marker as proxy for hepatic fibrosis (as transient elastography/Fibroscan is not available in this context). It uses the ratio of Serum Aspartate aminotransferase (AST) to the platelet count. [14,15]

$$APRI=\frac{AST(Upper\;Limit\;of\;Normal)}{Platelet\;count\;\left(10^{9}/L\right)}\times 100$$

**Table 1:** Treatment outcome definitions (adapted from the AASLD practice guidelines for Hepatitis C) and screening criteria for Hepatitis C risk factors in a primary health care-based program for management of chronic Hepatitis C, Karachi, Pakistan.

Definition of terms for Treatment Outcomes	Screening criteria involving risk factors for Chronic Liver Disease used in the MSF Hepatitis C Programme, Karachi, Pakistan
- Sustained Virological Response (SVR) 12: Absence of viremia 12 weeks post treatment as evidenced by a negative HCV RNA Quantitative PCR. (cured) - Relapse: Recurrence of viremia at 12 weeks post treatment after a negative end of treatment viral load. - Non-responder/ Treatment failure: Persistence of viremia at the end of treatment. - Stopped treatment: Medical decision to stop treatment either from complications of treatment or from decompensation. Patients are referred to tertiary care for further management. No viral load is taken after stopping treatment. - Loss-to-follow-up: Patients who missed follow-up consultation at least 60 days from the appointed date, despite tracing efforts. Died: Patients who died while enrolled to the programme.	- A patient with jaundice, ascites and/or elevated liver enzymes. - Husband or wife of an HCV patient - Treatment in the form of injections/infusions at local clinics with non-licensed personnel - Intravenous drug use - Major surgeries/dental procedures - History of blood transfusion - Mother is HCV-infected - History of previous jaundice - Female patient with HCV-infected child - History of incarceration - HIV-infection

The APRI score is used to prioritize patients in terms of treatment initiation: an APRI score of 1.0 is used as a threshold for treatment initiation; mainly due to resource constraints, which preclude the initiation of treatment for all CHC patients. Patients with an APRI score of ≥ 1 are prioritized and started on treatment after patient counseling concerning the disease, treatment, and lifestyle changes including family planning. Patients with certain conditions are assessed by the physician as ineligible for treatment or have the treatment deferred temporarily (see Table 2).

**Table 2:** Conditions for treatment ineligibility and treatment deferral in a primary health care-based program for management of chronic Hepatitis C, Karachi, Pakistan.

Patients with the following conditions are referred to a tertiary care facility for further management, and are ineligible to receive treatment from the programme.	Patients with the following conditions have treatment deferred temporarily and will be reassessed as indicated.
- Signs of decompensated liver disease on clinical examination (Child-Pugh score class B or C). These patients will be assessed individually. - Hemolytic anemia or autoimmune hepatitis - Renal impairment as evidenced by a Creatinine clearance below 50 ml/min. - History of hypersensitivity reaction to Ribavirin or its component. - Severe illness with WHO performance scoring of >2.	- Patients with co-infection—HIV/HBV. For HIV, ARV treatment is initiated prior to HCV treatment. The patient should be on ARV treatment for at least 3 months and have a viral load of <1000 copies/ml and a CD4 count of >50/μl. - Patients on treatment for TB should complete TB treatment first prior to initiating HCV treatment. - IV drug users not willing to adhere to treatment. - Patients with APRI scores below 1, they will be reassessed every 6 months. - Patients eligible for treatment but not willing to undergo family planning. - Patients with reversible causes of anemia. - Patients below 18 years old. - Pregnant patients and lactating mothers.

Treatment duration is based on HCV genotype. We use the same treatment combination for both treatment-naïve and treatment-experienced patients, using Sofosbuvir with weight-based Ribavirin.

Once on treatment, a patient comes for follow-up every month for drug refill, clinical and laboratory assessment, assessment of compliance to family planning advice, and lifestyle counseling sessions by patient support counselors. At the end of treatment, viral load is assessed. HCV viral load is also assessed twelve weeks after treatment completion to determine the sustained viral response (SVR) (see Table 2a). Patients with low-level viremia at the end of treatment also have viral load assessment 12 weeks after treatment completion for SVR assessment.

All diagnostics and treatment provided by MSF is free of charge. Based on the 2015 Sindh Provincial Hepatitis Programme estimate, the cost of Interferon treatment per patient is at 65 USD for conventional interferon and 800 USD for those on pegylated interferon.

### Population

All patients who were diagnosed in the programme over the course of 2015 were included in the study. For the analysis of treatment characteristics and treatment outcomes, a subset of adult patients who initiated treatment over the course of 2015 was selected.

### Variables and analysis

Data from the electronic Hepatitis C database was used to identify variables—patient’s sex; age; ID number; registration date; origin; APRI score; HIV and HBV status; TB workup (if applicable); previous HCV treatment history; genotype; pretreatment, end of treatment and 12-weeks post treatment viral loads; treatment initiation, completion and SVR dates; hemoglobin (Hb) determination over the course of treatment; ALT/AST determination pretreatment and end of treatment; and treatment outcomes. EpiData analysis software (version 2.2.2.183, EpiData Association, Odense, Denmark) was used for descriptive statistics describing the study population and treatment outcomes. Relative risks with 95% confidence intervals were calculated as measures of association for factors possibly associated with pretreatment attrition and adverse outcomes.

### Ethics approval

Permission to carry out the study was obtained from the Ethics Advisory Group of the Union. Local ethics approval was obtained from the National Bioethics Committee of the Pakistan Medical Research Council. The study fulfilled the exemption criteria set by the Ethics Review Board (ERB) of Médecins Sans Frontières (MSF), Geneva, Switzerland, for a-posteriori analyses of routinely collected data and thus did not require MSF ERB review. It was conducted with permission from the Medical Director of MSF-Operational Centre Brussels, Belgium.

## Results

### Characteristics at enrollment to the programme

For this study, records of 1089 patients were reviewed. Sixty two percent of patients were females, 52% belonged to the 25–44 years age group, and 70% originated from outside Machar Colony. Ninety percent were treatment naïve patients, and 372 (34%) patients had no significant liver fibrosis based on their APRI score index (see Table 3).

**Table 3:** Sociodemographic and characteristics of chronic Hepatitis C patients enrolled in a primary health care-based program for management of chronic Hepatitis C, Karachi, Pakistan; February to December 2015.

Category		Value (%) n = 1089
Sex	Male	409 (38)
	Female	675 (62)
	Not recorded	5 (<1)
Age (years)	5–18	14 (1)
	19–24	72 (7)
	25–44	561 (52)
	45–64	403 (37)
	65+	34 (3)
	Not recorded	5 (<1)
Patient’s Origin	Machar Colony	324 (30)
	Non Machar Colony	760 (70)
	Not recorded	5 (<1)
Previous hepatitis C treatment history	Treatment naïve	980 (90)
	Previously treated for Hepatitis C	109 (10)
APRI score	0.1–0.49	372 (34)
	0.5–0.99	278 (26)
	1.0–1.99	178 (16)
	2.0–2.99	63 (6)
	3.0–4.99	41 (4)
	5.0 +	13 (1)
	Not recorded	144 (13)
HIV status	Negative	972 (89)
	Positive	7 (1)
	Not recorded	110 (10)
Hepatitis B status	Negative	1017 (93)
	Positive	14 (1)
	Not recorded	58 (5)

APRI: AST to Platelet Ratio Index; HIV, Human Immunodeficiency Virus

**Fig 1:**
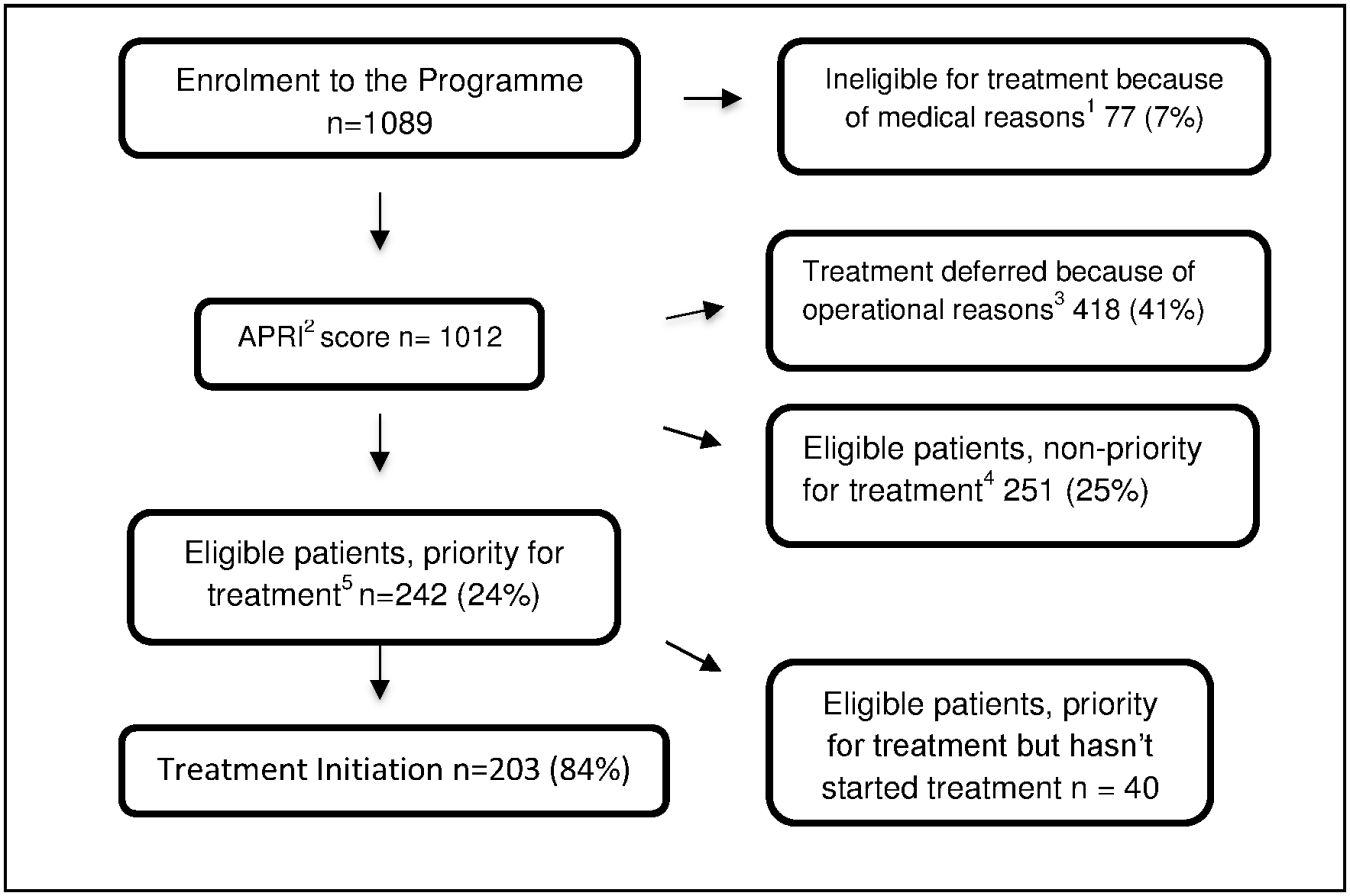
Flowchart for diagnosis and treatment prioritization among CHC patients in a primary health care-based program for management of chronic Hepatitis C, Karachi, Pakistan; February to December 2015. ^1^ Initial clinical assessment rules out patients with known medical conditions contraindicated for treatment initiation such as decompensated cirrhosis, hematologic diseases, severe renal insufficency. ^2^Aspartate aminotransferase (AST) to Platelet Ratio Index ^3^Patients with low APRI scores (0.1–0.49), with co-infection (TB, HIV, and Hepatitis B), Pregnancy and Lactation ^4^Patients with APRI score 0.5–0.99 ^5^Patients with APRI above 1.0

242 patients were considered high priority for treatment initiation (see Fig 1). Of these, 202 (83%) were ultimately placed on treatment, and 17% did not start treatment as scheduled, signifying at least a 9-month delay in treatment initiation and possibly complete pre-treatment loss-to-follow-up. Reasons for failing to initiate treatment included delayed family planning initiation (29 patients), patient’s unwillingness to start treatment (8 patients) and absent/ incorrect phone numbers for tracing (4 patients). Males, patients in the 25–44 years age group, and those with Genotype 1 infection were identified as being at higher risk for failing to initiate treatment as scheduled (see Table 4).

**Table 4:** Characteristics of patients in the priority list for Hepatitis C treatment and risk factors for delayed start of treatment in a primary health care-based program for management of chronic Hepatitis C, Karachi, Pakistan; February to December 2015.

Category		Treatment Status		RR (95% CI)	p-value
		Treated by MSF (%)	Not treated by MSF (%)		
		n = 202	n = 40		
Sex	Male	75 (76)	24 (24)	2.2 (1.2–3.9)	0.007
	Female	127 (89)	16 (11)	1	-
Patient’s Origin	Machar Colony	61 (85)	11 (15)	1	-
	Non Machar Colony	141 (83)	29 (17)	1.1 (0.6–2.1)	0.7
Age (years)	19–24	14 (93)	1 (7)	0.6 (0.1–4.0)	0.6
	25–44	79 (76)	25 (24)	2.0 (1.1–3.7)	0.01
	45–64	105 (88)	14 (12)	1	-
	65 +	4 (100)	0	NA	0.5
APRI	1.0–1.99	124 (82)	28 (18)	1	-
	2.0–2.99	44 (86)	7 (14)	0.8 (0.4–1.6)	0.4
	3.0–4.99	28 (85)	5 (15)	0.8 (0.3–2.0)	0.7
	5.0 +	6 (100)	0	NA	0.2
Genotype	1	10 (71)	4 (29)	6.7 (2.3–19.6)	0.002
	2	2 (100)	0	NA	1.0
	3	180 (96)	8 (4)	1	-
	Mixed genotype/not recorded	10 (26)	28 (74)	17.3 (8.6–35.0)	<0.0001

From the registration date to treatment initiation, there was a median delay of 72 days (IQR 37–100 days). There were no significant associations of the delay with APRI score, nor with location of diagnosis (whether internal or external HCV RNA PCR). From APRI eligibility to treatment initiation, there was a median time delay of 54 days (IQR 23–83 days) with the delay decreasing with increasing APRI scores.

### Treatment characteristics

**Fig 2:**
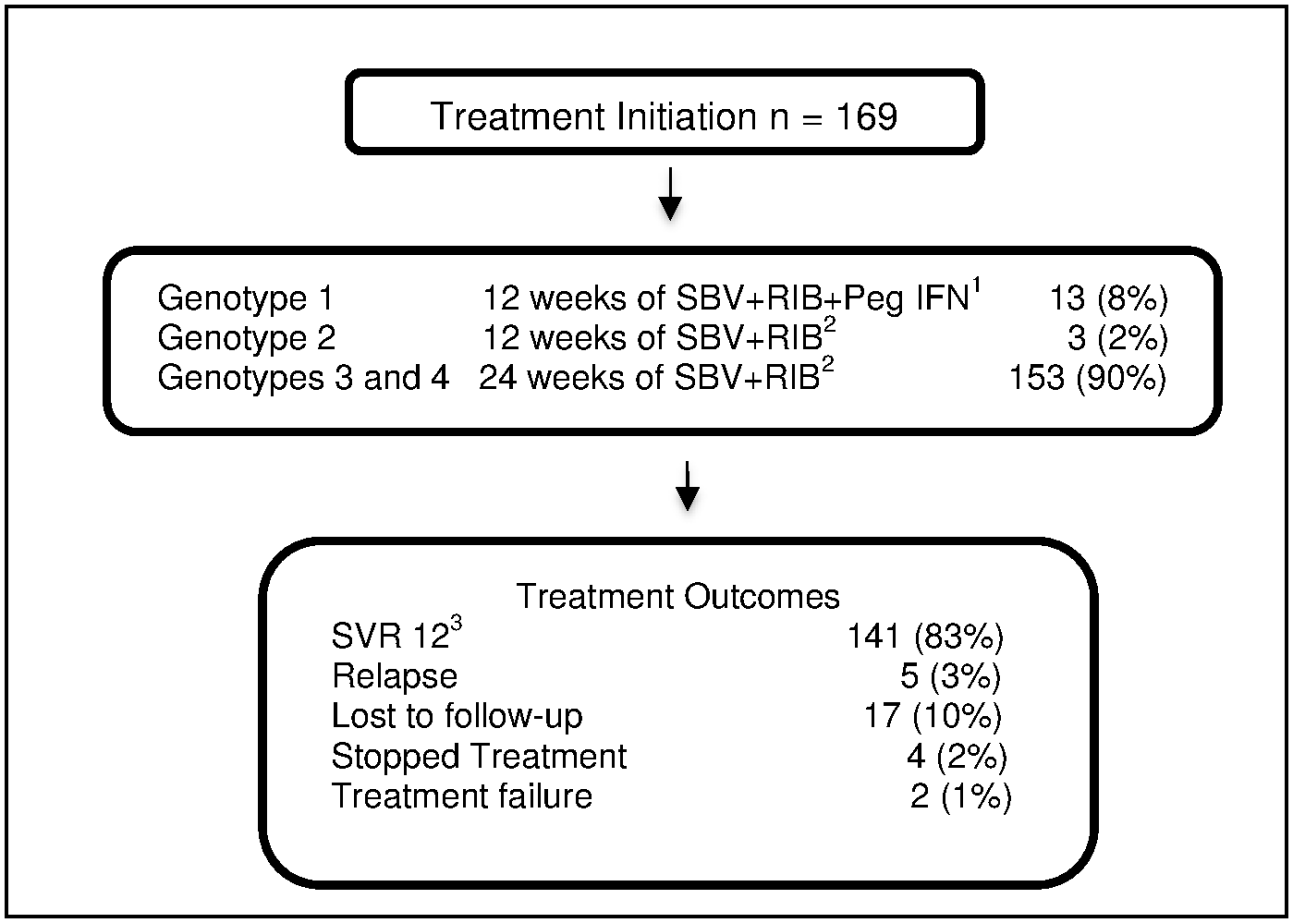
Flowchart for treatment among chronic Hepatitis C patients in a primary health care-based program for management of chronic Hepatitis C, Karachi, Pakistan; February to December 2015. ^1^ Sofosbuvir 400mg/day + weight-based Ribavirin 800–1200 mg/day + Pegylated interferon 180 μg/week Subcutaneous for 12 weeks ^2^ Sofosbuvir 400 mg/day +weight based Ribavirin 800–1200 mg/day ^3^ Sustained virological response (cure)

**Fig 3:**
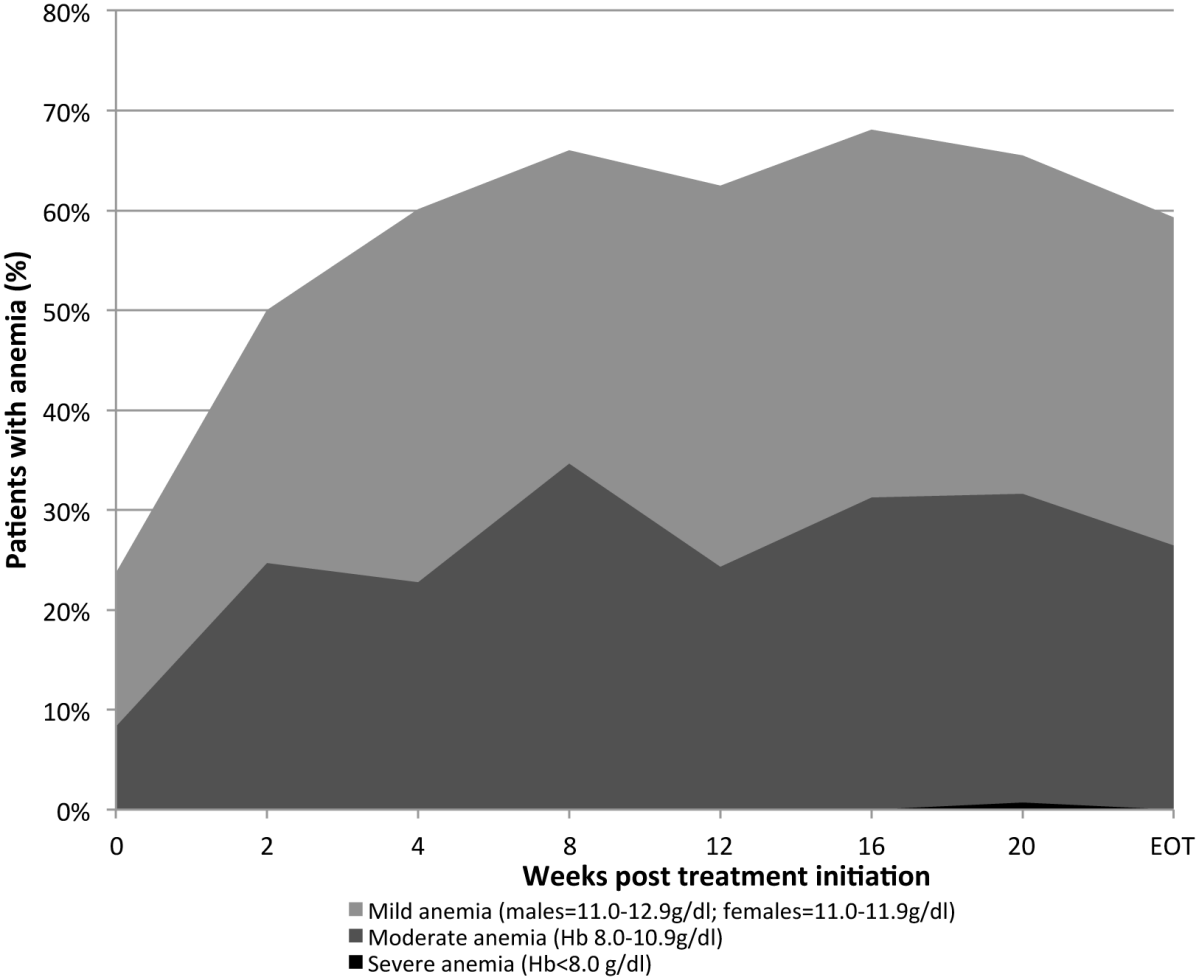
Percentage of chronic Hepatitis C patients who developed anemia while on treatment in a primary health care-based program for management of chronic Hepatitis C, Karachi, Pakistan; February to December 2015. ^1^ WHO. Hemoglobin concentrations for the diagnosis of anemia and assessment of severity.

One hundred sixty-nine patients were placed on treatment in 2015, and were followed up over their treatment course (Fig 2). Severe anemia was only observed in one patient over the 24-week treatment course, at week 20. Treatment was discontinued for 2 weeks, folate supplementation was continued and the patient was monitored for symptomatic anemia. Moderate anemia peaked at week 8 (Fig 3). For 53 patients (35%), the dose of Ribavirin was reduced as per recommendation, and patients were monitored for symptomatic anemia along with folate supplementation.

Median pretreatment ALT and AST for patients who initiated treatment was 77 and 84 IU/L, respectively. At the end of treatment, repeat ALT and AST were taken with median levels at 18 and 24 IU/L.

### Treatment outcomes

There were 141 (83%) patients achieving SVR at 12 weeks post treatment. Of these, 128 patients had genotype 3 infection (84% treatment success), and 10 had Genotype 1 infection (77% treatment success) (see Fig 2). Genotype, patient’s origin, previous treatment history and APRI scores had no significant associations with adverse outcomes.

Four patients stopped treatment because of medical reasons: a female patient had a positive pregnancy test 2 weeks after treatment initiation; 2 male patients stopped treatment because of hepatic decompensation while on treatment; and a female patient stopped treatment on her 2^nd^ week of treatment because of a hypertensive crisis.

Cases lost-to-follow-up were reviewed. Eight patients never came back for initial monitoring after treatment was initiated. Five patients had a negative end of treatment viral load but never came back for SVR determination. Reasons identified were absence/ incorrect contact number for tracing, and patients refusing to come back because they were out in their village, far from Karachi.

## Discussion

The novel approach of Hepatitis C management integrated into a primary health care setting using DAAs as treatment regimen yielded a treatment success rate of 83%. These results show how we have successfully simplified HCV management in a high-burden setting, and confirm under operational conditions the high efficacy of DAAs that has previously been reported in the controlled environment of clinical trials. [16–19]

The use of the Sofosbuvir-Ribavirin (SOF-RBV) treatment regimen in the Pakistani context is new, given that Sofosbuvir was only registered in Pakistan since November 2014. [20] Treatment outcomes for this setting have to our knowledge not yet been published. In this study, an integrated, decentralized model of care for HCV was effective, with loss-to-follow-up rates at 10%. The outcomes and treatment characteristics are in line with published evidence from other settings. The new all-oral treatment regimen, with the exception of GT1 (which requires pegylated IFN on top of the SOF-RBV combination), is highly tolerable with documented limited adverse events in clinical trials. [16–19] The VALENCE study, a multicenter Phase 3 trial in Europe showed that the SOF-RBV regimen for 24 weeks in GT3 patients had an SVR12 of 85%. [18] Conventional management relying on an IFN-based treatment regimen over a prolonged time period [10] resulted in general SVR rates of 42–93% for all genotypes, showing only moderate efficacy for the combination of pegylated IFN with Ribavirin in multiple randomized control trials. [19] A real life, retrospective study done at Queen’s Liver Center in Hawaii, on treatment outcomes as compared to the VALENCE study for GT3 patients on SOF-RBV regimen for 24 weeks showed SVR rates of 81%. [21] Two Indian studies gave initial real-life results from a treatment cohort of GT3 patients receiving 24-week of SOF-RBV, with SVR12 rates at 96–98% regardless of severity of disease or previous HCV treatment history. [22,23]

Hemolytic anemia associated with Ribavirin is commonly seen in patients on a SOF-RBV regimen. [18,21] An Hb reduction of 2.1 g/dl for a 24 week SOF-RBV treatment course for GT3 patients was observed in the VALENCE study with 6% of patients having Hb levels less than 11 g/dl at any point in the treatment. [18] Our treatment cohort recorded 25–35% of patients developing moderate anemia with Hb less than 11 g/dl at any time during the treatment course. Despite temporary discontinuation of treatment for one patient with severe anemia, SVR12 was achieved.

The following limitations were identified for this study. First, as the study reflected an interim analysis over the first 8 months of the programme, only a limited sample size for treatment outcomes could be obtained. This affected analysis in terms of establishing strong associations with adverse outcomes. Second, the database used for patients starting treatment over the course of 2015 did not allow linkage with systematic data concerning adverse events, and for the purposes of this study, only hemoglobin and liver enzyme levels were used for the monitoring of consequences of treatment. This has been remedied since, and future analyses will allow full reporting of adverse events. Third, data regarding patient’s previous HCV treatment history may have been inflated, as some patients were not able to present documents evidencing past HCV medical management; however, they were still included as patients with previous HCV treatment. Strengths included the operational nature of the study, ensuring that the results likely reflect the field conditions encountered by many operational actors; and adherence to the STROBE guidelines for reporting of observational data. [24]

Our study carried a number of implications. Seventy percent of patients did not belong to the geographic target population. Reasons identified were informal word of mouth publicity, popularity of the free treatment regimen with fewer side effects, and the scarcity of treatment options available in the local setting since many patients are undocumented limiting government health services. This was a challenge for the organization in terms of addressing the increasing gap from Machar Colony patients, who are the intended beneficiaries for the programme.

There is a median 53-day delay from treatment eligibility to treatment initiation, with an inverse correlation to APRI score; and one out of six patients had a delay in treatment initiation of at least 9 months. Lack of patient support, and familial or personal concerns regarding the treatment were linked to the delay, with some of the patients actually lost along the process. Males, mostly in the working age group (25–44 years) were at higher risk of treatment initiation delay, likely because they are breadwinners of the family and have to prioritize work over personal needs such as taking time off for treatment attendance.

The primary health care management of CHC showed good treatment outcomes. It ensured adherence to treatment through monthly follow-ups with patient support component, which likely contributed to the positive outcomes. Antiviral treatment is only one element of the comprehensive care for patients with CHC, and the primary care level may be the most appropriate setting to offer such comprehensive care to most people living with CHC. [25] In underserved areas of the United States the concept of task-shifting was developed, engaging mid-level healthcare practitioners in the management of CHC with DAAs with indirect supervision from a specialist: an SVR12 was achieved for 83% of GT3 patients in this treatment cohort. [26] Another study involving the Extension for Community Health Outcomes (ECHO) Model engaging PHC management of CHC in New Mexico, USA by training primary care providers showed overall SVR12 rates of 58.2%. [27] Overall, the encouraging programmatic outcomes suggest that the decentralized approach can serve as a model for other stakeholders contemplating a HCV programme.

Despite the efficacy of Sofosbuvir, its high cost limits its use in Pakistan, where the majority of patients belong to the lower income class. Currently, the innovator brand Sovaldi is sold at 1800 USD per treatment course for GT3 HCV infection. [20] However, the government has made steps in policy-making allowing for better competition among the pharmaceutical companies manufacturing Sofosbuvir, given that Pakistan is one of the high-burden countries for HCV. [28] The inclusion of the Sofosbuvir-based treatment regimen in the recently published National Guidelines for HCV in Pakistan [29] will pave the way for its widespread use by clinicians, and hopefully in the long run, influence policy makers for the inclusion of DAAs at both provincial and national levels. Such DAAs should not be limited to the Sofosbuvir-based treatment regimen: newer, more effective DAAs are in the pipeline, and some are in the process of being registered in Pakistan. At the time of writing, the programme had already incorporated Daclatasvir in the treatment regimen, offering a better safety profile and thus likely improving patient adherence.

## Conclusion

Hepatitis C management in a programmatic approach using an integrated decentralized model of care in a primary healthcare setting, using direct-acting antivirals, produces treatment outcomes comparable to clinical trials done for Sofosbuvir-based treatment regimens.

## Supporting information

S1 Checklist STROBE statement-checklist of items that should be included in reports of observational studies. (DOC)

S1 Dataset MSF Hepatitis C programme patients’ database from February–December 2015. (XLS)

S1 Protocol Primary health care management of chronic Hepatitis C in Karachi, Pakistan. (DOCX)
