# Correction: Management of chronic Hepatitis C at a primary health clinic in the high-burden context of Karachi, Pakistan

**DOI:** 10.1371/journal.pone.0180286

**Published:** 2017-06-22

**Authors:** Yuely A. Capileno, Rafael Van den Bergh, Dmytro Donchuk, Sven Gudmund Hinderaker, Saeed Hamid, Rosa Auat, Gul Ghuttai Khalid, Razia Fatima, Aashifa Yaqoob, Catherine Van Overloop

The third author’s name is spelled incorrectly. The correct name is Dmytro Donchuk. The correct citation is: Capileno YA, Van den Bergh R, Donchuk D, Hinderaker SG, Hamid S, Auat R, et al. (2017) Management of chronic Hepatitis C at a primary health clinic in the high-burden context of Karachi, Pakistan. PLoS ONE 12(4): e0175562. https://doi.org/10.1371/journal.pone.0175562
