# Supplementary material for: Management of chronic Hepatitis C at a primary health clinic in the high-burden context of Karachi, Pakistan
Source: PLoS One. 2017 Apr 27;12(4):e0175562. doi: 10.1371/journal.pone.0175562 (PMC5407611; doi:10.1371/journal.pone.0175562)
Supplement: S1 Protocol — (DOCX) [file pone.0175562.s003.docx]

**Primary Health Care (PHC) Management of Chronic Hepatitis C in Karachi, Pakistan**

Investigators

Yuely A Capileno^1^

Rafael Van den Bergh^1^

Dmytro Donchunk^1^

Rosa Auat^1^

Gul Ghuttai Khalid^1^

Sven Gudmund Hinderaker^2^

Razia Fatima^3^

Aashifa Yaqoob^3^

Saeed Hamid^4^

Institutions

1. Medecins sans Frontieres - Brussels
2. International Union Against Tuberculosis and Lung Disease, Paris, France; and the University of Bergen, Norway
3. National TB Control Program, Pakistan
4. The Aga Khan University and Hospital

Corresponding Author

Yuely A Capileno, Medecins sans Frontieres-Brussels

**BACKGROUND**

Hepatitis C Virus (HCV) infection is an urgent global health concern. The World Health Organization (WHO) estimates that more than 185 million people are infected with HCV. Transmission is blood-borne, occurring through unsafe injection practices, inadequate sterilization of instruments, blood transfusion, sexual transmission, and mother-to-child transmission.^1^ It is known that chronic Hepatitis C develops into cirrhosis and hepatocellular carcinoma, and approximately 350,000 people were estimated to die from these complications annually.^2^

The prevalence of HCV infection varies worldwide. The Middle East and North African region registers one of the highest prevalence of HCV, including Egypt and Pakistan.^3^ In 2014, thirty-one countries were identified to contribute 80% of the total viraemic infections worldwide.^4^ China, Egypt, Pakistan, Nigeria, India and Russia together account for more than half of the viraemic infections. ^4^

Pakistan is a lower middle-income country with a population of approximately 180 million.^5^ In 2014, an estimated adult anti-HCV prevalence of 6.7% with adult viraemic prevalence at 5.8% was reported in Pakistan. ^4,5^ The HCV prevalence in the general adult population among the four provinces of Pakistan varies, with Punjab having the highest prevalence at 6.7%, followed by Sindh at 5.5%, Balochistan at 1.5%, and Khyber Pakhtunkhwa at 1.1%.^6^ A recent review of HCV seroprevalence among the general population in Pakistan showed a 40% increase as compared to 2010 data.^7^

Ali et al., in their study on the risk factors for HCV in Pakistan identified unsterilized needles in healthcare setting, receipt of blood and blood products, occupational risks for healthcare workers, intravenous drug use, spousal transmission and shaving by barbers as the major drivers of HCV infection.^5^ Adnan et al., in their study on the comparison of the need and supply of syringes for the therapeutic injections in Pakistan have cited literature of the therapeutic use of injections being commonly sought in Pakistan, and often unnecessary.^8^ Most HCV infections in Pakistan are of genotype 3 (69.1%), followed by genotypes 1 (7.1%), 2 (4.2%) and 4 (2.2%).^5^

Querishi et al., in the 2013 audit of the Hepatitis B and C National Programme gathered 7754 cases of Hepatitis C receiving treatment. The Prime Minister’s programme for the Prevention and Control of Hepatitis viral infections was launched in Pakistan for 5 years from 2005 to 2010 to support treatment of Hepatitis B and C for patients who could not afford the treatment due to high cost of medicines and diagnostics, along with promoting preventive interventions.^9^ The national treatment protocol recommends 3-million unit interferon three times a week subcutaneously for 6 months together with antiviral Ribavirin twice a day (weight <70kg) for genotype 2 and 3 infections. For all other genotypes, either pegylated or conventional interferon is recommended for 12 months.^9^ From their audit, only 3440 cases completed the 6-month interferon therapy, while the rest were lost to follow-up and excluded from the final analyses. Of these, 1686 cases had HCV PCR results and were used to calculate the end of treatment response. Treatment response was at 67% for end of treatment viral clearance^9^. This programme is conventionally offered through specialized, tertiary care-level governmental hospitals.

Médecins Sans Frontières (MSF), an international medical humanitarian organization together with-a local nongovernmental organization SINA, operates a primary health clinic in Machar Colony, one of the biggest slums in Karachi. In 2013, the MSF team in Machar Colony conducted an assessment into the HCV burden in Machar Colony, and identified a high prevalence of key risk factors of HCV infection (high rate of therapeutic injections, intravenous drug use reports), and recommended to initiate HCV testing and treatment activities. At the same time, over the course of 2013 and 2014, introduction of direct-acting antivirals (DAAs) for treatment of Chronic Hepatitis C (CHC) dramatically changed the standard of care and allowed further simplification of HCV treatment delivery.

The MSF Hepatitis C programme started in February 2015. Between February 2015 and March 2016, 1600 patients with CHC have been enrolled in care, and treatment was initiated for 311 patients. To ensure access to HCV treatment for the residents of Machar Colony, the MSF programme delivers HCV Treatment in a primary care setting,, integrated in a primary health care center, using simplified diagnostic and treatment protocol. The programme has currently been operating for over a year.

Available data on CHC treatment in Pakistan describes outcomes of Interferon (IFN)-based therapy, but no published data on programme outcomes of CHC treatment using IFN-free DAAs regimens was available as of May 2016; and provision of HCV care integrated in primary health care was likewise poorly documented. In this work, we thus aim to present an interim analysis of treatment outcomes of patients enrolled in the HCV programme of the MSF Machar Colony Clinic.

Specific objectives:

Among Chronic Hepatitis C patients enrolled for care at the MSF Clinic in Karachi between February 2015 – December 2015, specific objectives are:

1. To describe the sociodemographic and clinical characteristics of patients enrolled in the programme, and the proportions of patients initiated on treatment, stratified by HCV treatment history and disease severity,
2. To document the treatment characteristics of patients initiated on therapy,
3. To present treatment outcomes of patients, stratified by HCV treatment history and disease severity,
4. To document the characteristics of patients with adverse treatment outcomes.

**METHOD**

Study Design

This is a retrospective cohort study of CHC patients enrolled in a primary health care clinic in Karachi, Pakistan

Study Setting

-Karachi is the capital city of the Province of Sindh, and as of 2013, has an approximate population of 23.5 million. It is the main seaport and financial center of Pakistan.^10^

The MSF Hepatitis C programme

Patients attending the MSF clinic and meeting the screening criteria (see Annex ) are screened using a rapid diagnostic test for HCV antibody. Once tested positive, HCV infection is confirmed through qualitative PCR. Positive patients are then enrolled for care and assessed for treatment eligibility using the aminotransferase/platelet ratio index (APRI) score, a noninvasive serum marker as proxy for hepatic fibrosis. It uses the ratio of Serum Aspartate aminotransferase (AST) to the platelet count. ^11^

AST (Upper Limit of Normal of AST)

APRI = x 100

Platelet count (10^9^/L)

The APRI score is used to prioritize patients in terms of treatment initiation: an APRI score of 1.0 is used as a threshold for treatment initiation; mainly due to resource constraints, precluding the initiation of treatment for al CHC patients. Patients with an APRI score of ≥ 1 are prioritized and started on treatment after patient counseling concerning the disease, treatment, and lifestyle changes including family planning.

For the following categories of patients, treatment is deferred:

- Patients with APRI scores below 1 are reassessed every 6 months,
- Patients with reversible causes of anemia,
- Pregnant patients and lactating mothers,
- Patients below 18 years old,
- Patients with co-infection – HIV/ HBV. For HIV, ARV treatment is initiated prior to HCV treatment. The patient should be on ARV treatment for at least 3 months and have a viral load of <1000 copies/ml and a CD4 count of >50/µl,
- Patients on treatment for TB should complete TB treatment first prior to initiating HCV treatment,
- Patients eligible for treatment but not willing to undergo family planning,
- IV drug users not willing to adhere to treatment

Patients with the below conditions are advised to be referred to tertiary care centers for specialized management.

- Signs of decompensated liver disease on clinical examination (Child-Pugh score class B or C). These patients will be assessed individually.
- Hemolytic anemia or autoimmune hepatitis
- Renal impairment as evidenced by a creatinine clearance below 50 ml/min.
- History of hypersensitivity reaction to Ribavirin or its component
- Severe illness with WHO performance scoring of >2.

Treatment is based on viral genotype. Same treatment protocol is used for both treatment-naïve and treatment-experienced patients, using the DAA- Sofosbuvir with weight-based Ribavirin.

- The treatment regimen for genotype 1 infection involves a triple regimen with Pegylated interferon 180 µg/week SQ + Sofosbuvir 400mg OD + weight-based Ribavirin (800-1200mg/day) to be given for 12 weeks.
- For genotype 2, a dual regimen with Sofosbuvir 400mg OD + weight-based Ribavirin (800-1200mg/day) to be given for 12 weeks is used.
- For Genotypes 3 and 4 infection, dual regimen with Sofosbuvir 400mg OD + weight-based Ribavirin (800-1200mg/day) to be given for 24 weeks is used.

Once on treatment, a patient comes for follow-up every month for monthly drug supply, assessment of adverse effects, compliance to family planning advice, and lifestyle counseling sessions by patient support counselors.

At the end of treatment, viral load is assessed. If found to be negative, the patient is reassessed for HCV viral load twelve weeks after treatment completion, to assess whether a sustained virological response (SVR) has been achieved.

Study Population

All patients enrolled in the Hepatitis C Programme of MSF Primary Health care Center in Karachi, Pakistan from February 2015 to December 2015 will be included in this study.

We will exclude pediatric patients aged less than 18 years because of a different treatment regimen used which is not included in the protocol mentioned above.

Data Variables, Sources of Data, and Data Collection

Source of data will be the electronic Hepatitis C database of all patients enrolled to the programme.

Variables will be identified according to the specific objectives as follow:

Specific Objective 1: (*To describe the sociodemographic characteristics of patients enrolled to the programme stratified by previous HCV treatment history and APRI score.* Data variables would include:

- Patient’s ID number
- Registration date
- Age
- Sex, (M/F/not determined)
- Origin (Machar colony/ non-Machar colony/ not determined)
- Genotype (1/2/3/4/mixed/not determined)
- APRI score
- Previous HCV treatment (treatment naïve/ treatment experienced/not recorded)
- Pretreatment viral load
- HBV status (positive/negative/not determined)
- HIV status (positive/negative/not determined)
- Started on treatment
- If not started on treatment: reason for not starting

Specific Objective 2: (*To document the treatment characteristics of patients initiated on therapy*) Variables for this would include

- Patient ID number
- Hb level
- Date of Hb measurement
- Creatinin clearance (CrCl)
- Date of CrCl measurement
- Adverse events during treatment
- Date of adverse event during treatment
- Outcome of adverse event during treatment (referral, treatment discontinuation)

Specific Objective 3: (*To present treatment outcomes of patients stratified according to HCV treatment history and severity of the disease based on APRI score)* Variables for this would include

- Patient’s ID number
- APRI score
- previous HCV treatment (treatment naïve/ previously treated/not recorded)
- pretreatment viral load
- treatment initiation and completion datestreatment outcomes (SVR/ relapse/ treatment failure/ stopped treatment/died/ lost to follow-up and not recorded)

Specific Objective 4: (*To present the clinical characteristics of patients with adverse treatment outcomes.*  Data variables would include:

- Patient’s ID number
- Registration date
- Age
- sex, (M/F/not recorded)
- genotype (1/2/3/4/mixed/not recorded)
- APRI score
- previous HCV treatment (treatment naïve/ previously treated/not recorded)
- pretreatment viral load
- treatment initiation and completion dates
- treatment outcomes (SVR/ relapse/ treatment failure/ stopped treatment/died/ lost to follow-up and not recorded)

Definition of terms for treatment outcomes is as follows:

Cured: those who attained SVR12 (defined as 12 weeks after treatment completion).

Relapse: those who completed treatment and had negative HCV viral load result at completion of treatment, but were not able to reach SVR.

Treatment failure: those who completed treatment, but with a persistent positive viral load

Stopped treatment: those patients who had to discontinue treatment due to complications of decompensation or complications from treatment

Lost to follow-up: those patients, who missed a follow-up session and could not be traced despite efforts for 14 days from the date of scheduled follow-up

Not recorded: no information about the patient

Variables required to this study will be extracted from the electronic database and analyzed.

Analysis and Statistics

Data will be analyzed using Epidata analysis software (version 2.2.2.183, EpiData Association, Odense, Denmark). The cascade of management pathway of study population enrolled will be depicted using a flowchart with frequencies and proportions at every step. Descriptive statistics will be used to describe the study population. Categorical variables will be summarized in terms of frequencies and proportions and continuous variables will be summarized in terms of mean (standard deviation) or median (interquartile range) as appropriate. Relative risk with 95% confidence interval will be calculated as a measure of association to describe treatment outcomes. The detailed plan of analysis including dummy analytic shells is annexed.

**ETHICS APPROVAL**

Ethics review will be sought from the Ethics Advisory Group of International Union Against Tuberculosis and Lung Disease, Paris, France and the National Bioethics Committee of Pakistan. – Pakistan Medical Research Council (PMRC).

Data Confidentiality

Data included in this analysis does not include patient identifiers. Data will be entered in a designed format based on the information recorded from the electronic database. Data will be kept for five years in a password-protected computer.

Specific patient benefits

The participants in this study will not receive any direct benefits, but later patients in the program may benefit from the findings, as this regimen is new in this primary health care setting.

Community participation and benefits

As we are offering a novel model for HCV management in the Pakistani context, results from this study could be used as an example to show the local community, better options for HCV management.

Feedback and dissemination of results

Results of the study will be shared with interested stakeholders for possible lobbying in policy change. The study will also be presented to the scientific community for critique, and will be submitted in an open access, peer-reviewed journal.

Implications for policy and practice

Results of the study will give us a better understanding of the programme outcomes and the feasibility of integration of HCV care at primary health care level.

Collaborative partnerships

The research will be conducted by staff of the MSF OCB – Karachi Project in collaboration with Coordination team from MSF-OCB Islamabad and with the approval of the Medical and Operational departments of the MSF OCB (Operational Centre Brussels).

**REFERENCES**

1. World Health Organization. Guidelines for the screening, care and treatment of persons with Hepatitis C infection. ISBN 978 92 4 154875 5; Paris, France: WHO, 2014
2. Khayriyyah Mohd Hanafiah, Justina Groeger, et. al. Global Epidemiology of Hepatitis C Virus Infection: New Estimates of Age-Specific Antibody to HCV Seroprevalence. *Hepatology* 2013; 57 (4): 1333-1342
3. Yousra Mohamoud, Suzanne Riome, Laith J. Abu Raddad; Epidemiology of the Hepatitis C virus in the Arabian Gulf countries: systematic review and meta-analysis of prevalence. *International Journal of Infectious Disease.* 2016; <http://dx.doi.org/10.1016/j.ijid.2016.03.012>
4. Erin Gower, Chris Estes, et.al. Global Epidemiology and genotype distribution of the Hepatitis C virus infection. *Journal of Hepatology* 2014; 61: S45-S57
5. Syed Asad Ali, Rafe MJ Donabue, Huma Qureshi, Sten Vermund. Hepatitis B and Hepatitis C in Pakistan: prevalence and risk factors. *International Journal of Infectious Diseases* 2009; 13 (1): 9-19
6. Muhammad Umar, Mohammad Bilal. Hepatitis C, a mega menace: A Pakistani perspective. *Journal of Pioneering Medical Studies* 2012; 2 (2): 68 – 72
7. Muhammad Umer, Mazhar Iqbal. Hepatitis C Virus prevalence and genotype distribution in Pakistan: a comprehensive review of recent data. *World Journal of Gastroenterology* 2016; 22(4): 1684 – 1700
8. Adnan Ahmad Khan, Momina Saleem, Huma Qureshi, et al., Comparison of need and supply of syringes for therapeutic injection use in Pakistan. Journal of Pakistan Medical Association 2012; 62:1149-1153
9. Huma Qureshi, Bile Khalif Mohamud, Syed Ejaz Alam, et.al. Treatment of Hepatitis B and Hepatitis C through national programme – an audit. *Journal of Pakistan Medical Association* 2013; 63: 220 – 224
10. Karachi. <https://en.wikipedia.org/wiki/Karachi>. Accessed: 25/03/2016
11. Chun-Tao Wai, Joel Greenson, Robert Fontana, et. al. A Simple noninvasive index can predict both significant fibrosis and cirrhosis in patients with chronic hepatitis C. *Hepatology* 2003; 38 (2): 518 – 526
12. Zhong-hua Lin, Yong-ning Xin, Quan-jiang Dong, et. al. Performance of the Aspartate Aminotransferase-to Platelet Ratio Index for the Staging of Hepatitis C-Related Fibrosis: An Updated Meta-Analysis. *Hepatology* 2011; 53 (3): 726 – 736
